# Comparison of intra-ocular pressure changes with liquid or flat applanation interfaces in a femtosecond laser platform

**DOI:** 10.1038/srep14742

**Published:** 2015-10-06

**Authors:** G. P. Williams, H. P. Ang, B. L. George, Y. C. Liu, G. Peh, L. Izquierdo, D. T. Tan, J. S. Mehta

**Affiliations:** 1 Tissue Engineering and Stem Cell Group, Singapore Eye Research Institute, Singapore; 2 Singapore National Eye Centre, Singapore; 3 Department of Ophthalmology, Yong Loo Lin School of Medicine, National University of Singapore, Singapore; 4 Ophthalmology Academic Clinical Program, Duke-NUS Graduate Medical School, Singapore; 5 Department of Clinical Sciences, Duke-NUS Graduate Medical School, Singapore; 6 Oftalmosalud Instituto de Ojos, Lima, Peru

## Abstract

Cataract surgery is the most common surgical procedure and femtosecond laser assisted cataract surgery (FLACS) has gained increased popularity. FLACS requires the application of a suction device to stabilize the laser head and focus the laser beam accurately. This may cause a significant escalation in intra-ocular pressure (IOP), which poses potential risks for patients undergoing cataract surgery. In this study we aimed to assess the effect of the Ziemer LDV Z8 femtosecond cataract machine on IOP. We demonstrated through a porcine model that IOP was significantly higher with a flat interface but could be abrogated by reducing surgical compression and vacuum. Pressure was lower with a liquid interface, and further altering angulation of the laser arm could reduce the IOP to 36 mmHg. A pilot series in patients showed comparable pressure rises with the porcine model (30 mmHg). These strategies may improve the safety profile in patients vulnerable to high pressure when employing FLACS with the Ziemer LDV Z8.

Femtosecond laser assisted cataract surgery (FLACS) with lens implantation offer potential advantages over conventional surgery including a controlled, precise means of creating a capsulotomy in order to remove the lens material and a reduction in power needed to complete phacoemulsification following femtosecond laser lens fragmentation^1^,^2^,^3^.

Femtosecond lasers rely on the use of docking systems in order to stabilize the eye and target laser energy, but inherently generate an increase in intra-ocular pressure (IOP) that in turn can potentially lead to optic neuropathy^4^. Docking systems comprise a flat or curved applanation interfaces bought in to direct contact with the cornea, or a liquid based system, to minimize distortion, with a liquid chamber that is attached to the laser. Either approach relies on the surgeon inducing mild compression on the globe prior to the applanation process and prior to the generation of machine vacuum. In turn suction stabilizes the eye before the laser is activated. It is crucial that suction breaks do not occur during surgery and therefore the surgeon must ensure adequate vacuum is maintained but with the minimal amount of inducible compression.

Several femtosecond laser platforms use curved or liquid applanation systems in order to undertake lens capsulotomy and fragmentation^2^,^3^. *In vivo* studies using real-time IOP cannulation have shown that lower pressures are generated with curved applanation systems such as the Visumax (Carl Zeiss) compared to flat applanation systems (e.g. earlier LDV (Ziemer) models), used for LASIK flap creation^5^,^6^. During femtosecond cataract surgery human studies have demonstrated that the intra-ocular pressure at the stage of suction docking were less than 30 mmHg with liquid interface systems such as Catalys^7^,^8^ and over 40 mmHg with the Victus platform^9^.

The Femto LDV Z8 (Ziemer Ophthalmic Systems AG, Port, Switzerland) is a new cataract platform that like its predecessor, the Femto LDV Z6 for LASIK flap formation, employs a low energy high frequency system, potentially reducing wound healing and scar tissue formation^10^. The Z6 model facilitates corneal applications using a flat interface while the Z8 model has a liquid patient interface for cataract surgery or a flat interface for the cornea module. Both are mobile devices, which have a moveable arm with counterweight to control the laser head.

To our knowledge, the intra ocular pressure generated with the same Ziemer laser platform, with flat vs. liquid interfaces, is unknown. Furthermore comparisons between real-time changes during the different stages of Femto-cataract process have not been described. The aim of this study was to determine the effect of the Ziemer LDV Z8 Femtosecond liquid patient interface laser platform on intra-ocular pressure (IOP) during lens fragmentation and capsulotomy in comparison to the Z6 flat applanation system.

## Results

### Comparisons between the Z8 liquid Interface and Z6 flat applanation system

**Figure 1:**
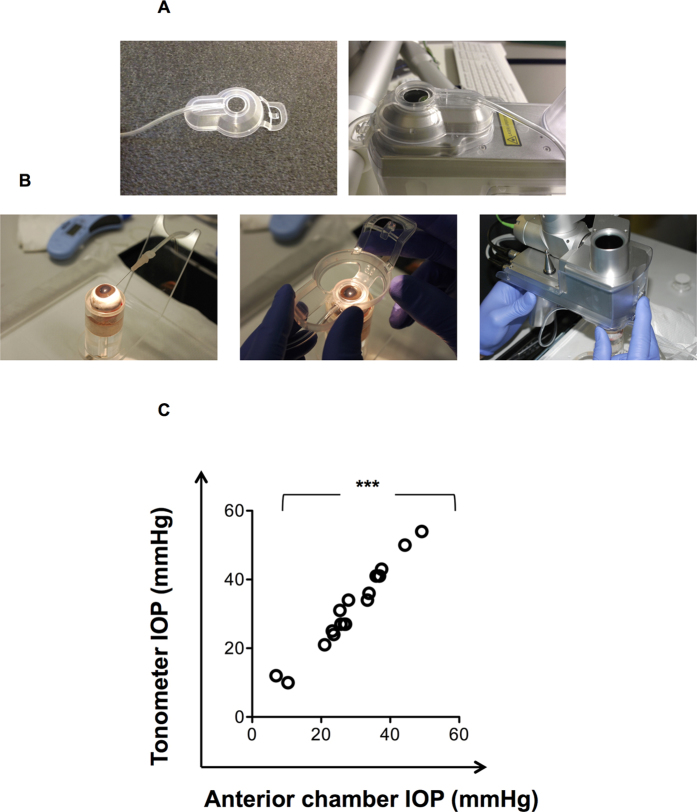
Set up and calibration of intra-ocular pressure measurement with the Ziemer Z8 (liquid) and Z6 (flat) applanation systems. Colour photographs showing the liquid and flat applanation systems employed for the Ziemer LDV Z8 ((**A**), upper left panel) and LDV Z6 ((**A**), upper right panel) respectively. Note the opening in the liquid interface compared to the flat applanation device. The system set-up including intra-ocular 30-g measuring device insertion posterior to the applanation device and angulation in to the anterior chamber is shown in (**B**) (lower left panel). Liquid-interface applanation (Panel (**B**), centre) prior to and after vacuum/ liquid filling/suction with the Ziemer LDV Z8 ((**B**), lower right panel) are also demonstrated. Correlation of intra-ocular IOP between anterior chamber cannulation (LabChart) and a Reichert Tonopen was undertaken following calibration and prior to commencement of surgery (n = 18) (Panel (**C**)). Spearman’s correlation. p < 0.0001***.

**Figure 2:**
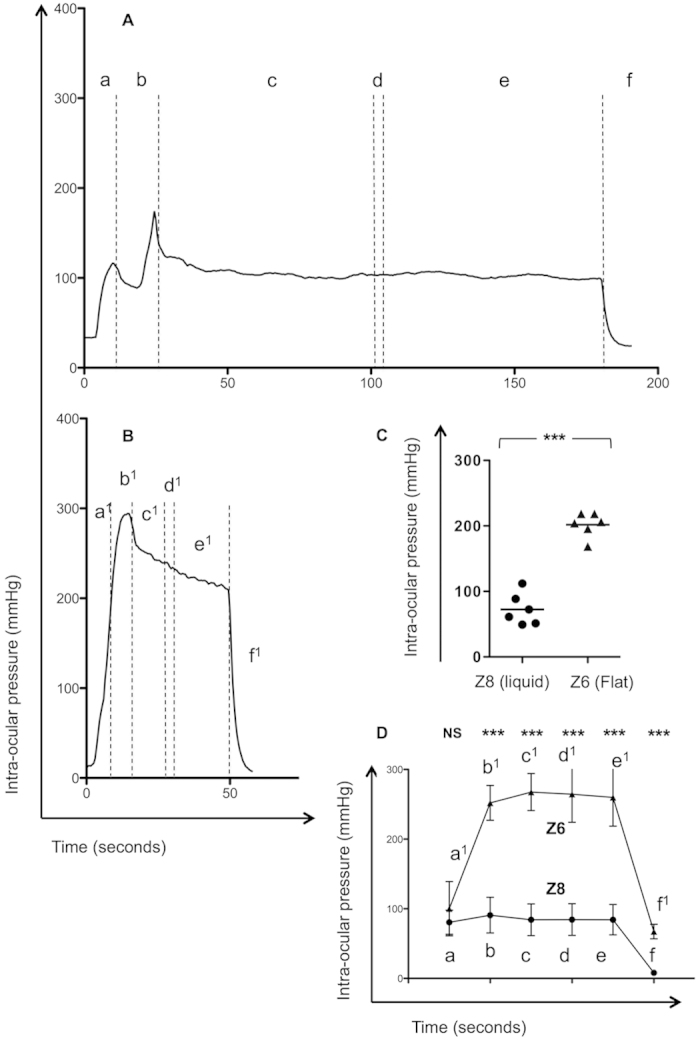
Fluctuation of IOP and time course of fragmentation/capsulotomy with a liquid interface (Z8) or LASIK flap cutting with a flat interface (Z6) with a Ziemer femtosecond laser. Representative timeline for the liquid (Z8) and flat (non-liquid, Z6) interface systems. Panel (**A**) shows the time-course from docking (applanation and vacuum) and the different stages of Femto-fragmentation and Capsulotomy for the Z8 liquid interface and panel (**B**) the Z6 Flat Interface for LASIK flap cutting. The sequence is: Time ‘0’; a Applanation* (a, Liquid patient interface or a1, Flat applanation attachment to the globe); b Vacuum followed by liquid filling and attachment of laser hand piece, corresponding to the peak IOP (Z8) or b1 Vacuum alone for the flat interface (Z6); c Lens Fragmentation (c1 Lamella cut); d Pause (d1 Pause); e Lens Capsulotomy (e1 Side cut); f /f1 Cessation of suction and normalization of IOP. Panel (**C**) shows the average IOP for the Z8 and Z6 during the whole procedure and (**D**) shows the schematic average IOP (mmHg) for the Z8 liquid interface (closed triangles) and the Z6 flat interface (closed circles). Comparison was by the unpaired t test (n = 12) (p < 0.05 significant). *NB - the globe was compressed with the applanation device prior to the generation of adequate suction. p < 0.001***.

The set up of the instrumentation is shown in Fig. 1A/B. The average IOP was 74.2 mmHg (±18.31) during the procedure with the Z8. Representative plots of the time course and average IOP during the procedure illustrate the different profiles seen with fragmentation/capsulotomy (Fig. 2A,B). Sub-group comparison also revealed that the average IOP was significantly lower with the Z8 fragmentation/capsulotomy 72.50 mmHg (±24.2) than with Z6 LASIK flap creation, 201.9 mmHg (±18.55) (p < 0.0001). The average IOP rise between platforms is shown in Fig. 2C. This was evident in every step of the procedure with exception of the initial docking stage a/a^1^ (Fig. 2D, Supplementary Table). A stable profile was observed during the laser cutting stages following a peak IOP at the end of the docking stage b/b^1^.

The average time taken to complete the procedure was 216.4 seconds(s) (±43.5 s) (n = 18) with the LDV Z8 platform. There was significant variation for undertaking different sized capsulotomies however, ranging from 180.0 s (±26.38) for a 4 mm capsule to 248.7 s (±26.23) for a 6 mm capsule (Supplementary Figure 1A) (p = 0.01, 4 vs. 6 mm p < 0.05). The greatest variation was seen for the time interval from docking to commencing fragmentation (74.96–126.2 s) and creation of capsulotomy (41.22–60.35 s). When comparing a sub-group of 5 mm capsulotomies (n = 6) with the Z6 platform (n = 6) the time taken to complete the LASIK procedure were predictably faster (56.07 s ± 2.96) than fragmentation/capsulotomy (220.5 s ± 46.81) (p < 0.0001) (Supplementary Table, Supplementary Figure 1B).

### Determining the effects of the hand piece with the Z8 and Z6 on IOP

We determined that three potential elements could affect IOP during the procedure: (1) the weight of the hand piece, (2) the compression on the globe by the surgeon during the procedure, and (3) suction generated through vacuum by either interface. Marked elevation in IOP occurred during applanation a/a^1^ (either with the flat applanation or liquid interface), followed by further escalation during suction, b/b^1^ (Fig. 2A,B). Typically during docking, compression was applied during the applanation stage prior to successful vacuum generation and then released.

In order to determine the influence of the hand piece weight, the IOP was measured with both Z8 and Z6 hand pieces without the application of suction. Briefly, the docking process was undertaken in exactly the same fashion as a complete procedure, i.e. with compression applied as normal (prior to achievement of b/b^1^ ‘suction’) and release of pressure.

**Figure 3:**
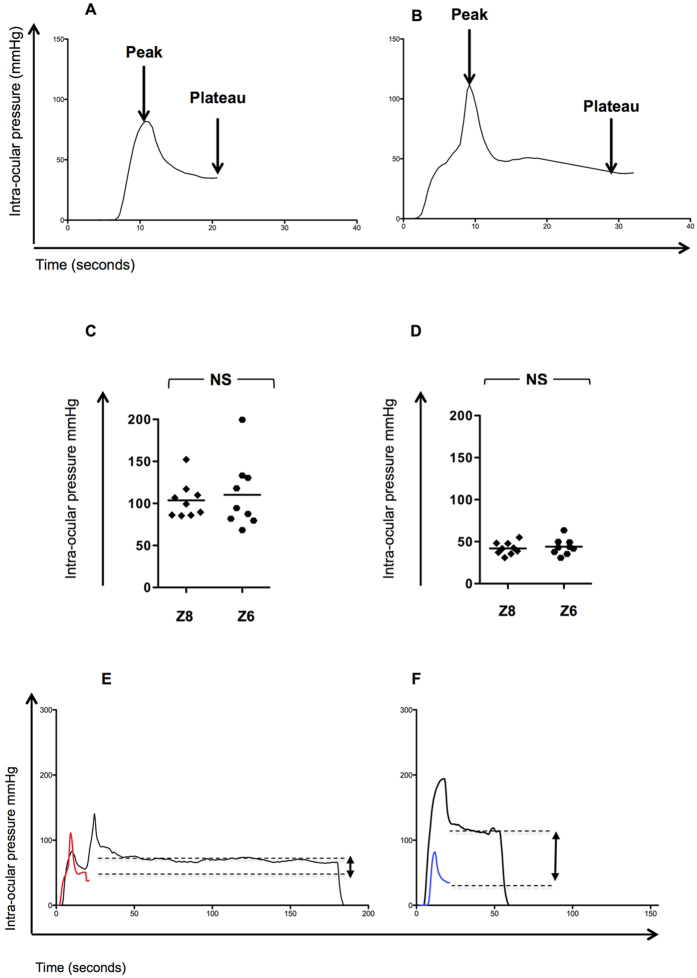
The effects of the Ziemer Z8 (liquid) and Z6 (flat) applanation laser hand piece weight on Intra-Ocular Pressure (IOP). The effect of handpiece weight on IOP as determined by simulating the docking process without applying suction (vacuum). In order to achieve this the applanation device was placed on the globe with commensurate pressure to normal docking but without suction. Initial pressure (peak) is normally required before vacuum takes effect and then relieved (plateau) where no surgeon compression took place. The maximal (peak) and minimal (plateau) intra-ocular pressure (IOP) seen during the docking process, in the absence of suction use or laser application are shown in (**A**) (Z8) and (**B**) (Z6). Differences in peak (**C**) and plateau IOP (**D**) are represented and comparison is by the unpaired t test (n = 18) (p < 0.05 significant). Representative plots demonstrating the difference between plateau IOP with suction (black, complete procedure NS = Not Significant timecourse) and without (colour) are shown in (**E**) (Z8) and (**F**) (Z6).

Representative examples of the maximal and minimal pressure effects without suction are shown in Fig. 3A,B. There was no difference seen in the peak IOP during docking (weight + compression, corresponding to a/a^1^) without the effect of suction for the Z8 platform or Z6 platform (103.6 mmHg ± 21.7 vs. 110.3 mmHg, ±40.7, p = 0.67; Fig. 3C). This was also observed during the plateau phase (c/c^1^), 41.98 mmHg (±7.4) and 43.9 mmHg (±9.61), p = 0.64 (Fig. 3D) and represents the contribution of the hand piece weight on IOP (n = 18). Representative examples compared to the IOP during Z8 and Z6 laser application are shown in Fig. 3E,F.

### The effects of suction and compression on IOP

#### Z8 liquid interface

The effects of suction for the Z8 liquid interface could be represented by the difference for the normalized IOP i.e. mean IOP during c to e - (hand piece weight + surgical compression). The hand piece was held in a ‘neutral’ position when suction had been achieved and therefore did not require any further compression during the laser stages, negating its effect on IOP. This represents an effect of suction of 84.3-(42.1 + 0) and therefore approximates to 42.2 mmHg.

In an attempt to reduce the effect of suction on IOP further we undertook the procedure at a reduced vacuum of 300 mbar (n = 6), from 400 mbar. Results obtained showed no difference for overall IOP recorded using Z8 at 300 mbar compared to the standard setting of 400 mbar (82.3 ± 14.5, p = 0.42) and no differences in the individual stages (data not shown) although a recurring occurrence of suction loss for one eye.

#### Z6 flat interface

For the Z6, a higher initial pressure (up to 310 mmHg) was needed in order to facilitate adequate generation of suction through the flat applanation surface. The difference for the normalized IOP (suction and compression) equated to 223 mmHg (mean 267 mmHg for stages c^1^ to e^1^ - hand piece weight of 43.9 mmHg). We therefore determined the relative effects of residual surgical compression to avoid suction loss and suction itself with the flat interface on IOP.

**Figure 4:**
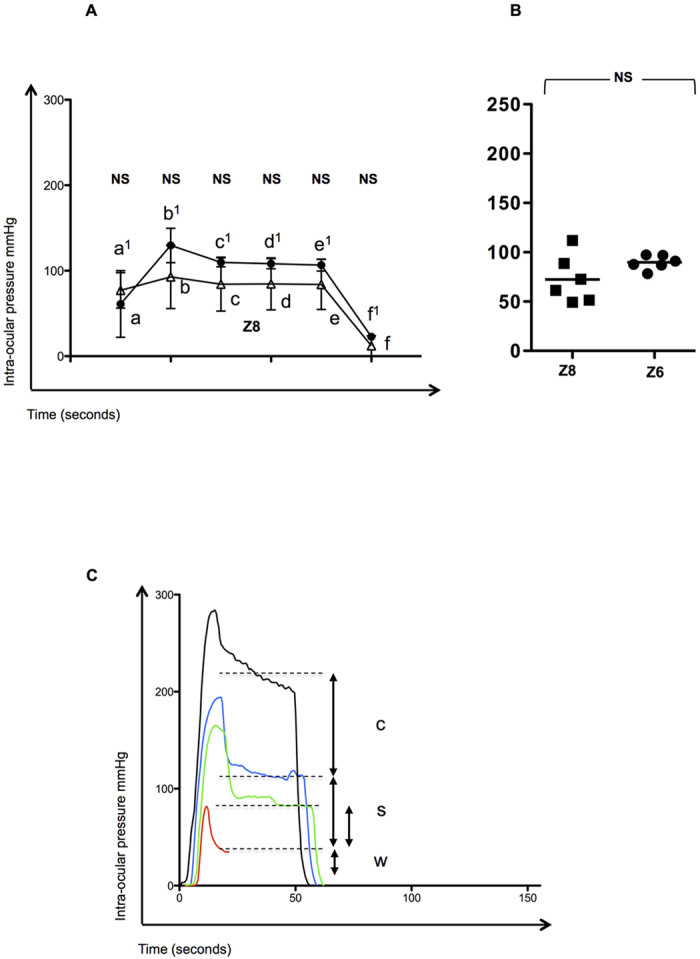
The effects of suction (vacuum) and surgeon compression on Intra-Ocular Pressure (IOP) during the Ziemer Z6 LASIK application. In order to determine the effect that vacuum and surgical compression contributed to IOP we attempted to reduce surgeon compression by avoiding post vacuum compression and by reducing mechanical suction. The effect of reduced compression on IOP is demonstrated for the Z6 laser (closed circles) at different stages of the procedure and comparisons with the Z8 (open triangles) are shown in Panel (**A**) and as an average (**B**). The sequence of the procedure is: Time ‘0’; a Applanation* (a, Liquid patient interface or a1, Flat applanation attachment to the globe); b Vacuum followed by liquid filling and attachment of laser hand piece, corresponding to the peak IOP (Z8) or b1 Vacuum alone for the flat interface (Z6); c Lens Fragmentation (c1 Lamella cut); d Pause (d1 Pause); e Lens Capsulotomy (e1 Side cut); f /f1 Cessation of suction and normalization of IOP. No significant suction losses (a potential concern with inadequate vacuum) occurred. Representative plots showing the differences in IOP during Z6 LASIK are shown in 4C by conventional compression (black), reduced compression (as outlined above, blue) alone and combined with reduced vacuum from 700 to 500 mbar (green). c = compression, s = suction and w = weight. The hand piece IOP (affect of weight alone) is shown for comparison (red). Comparison is by the unpaired t test (p < 0.05 significant). *NB - the globe was compressed with the applanation device prior to the generation of adequate suction NS = Not Significant.

First, the influence of this compression post suction was addressed by neutralizing surgeon compression. This approach resulted in an overall reduction in the IOP with the Z6 from 201.9 to 89.7 (±7.1), p < 0.0001. The average IOP from stages c^1^ to e^1^ averaged 108 mmHg by this method suggesting that the effect of compression was approximately 159 mmHg (267–108 mmHg) and suction generated through the Z6 flat interface equated to 64 mmHg (108–43.9 mmHg). No significant suction losses occurred and comparisons between reductions in compression alone with Z8 interface are shown in Fig. 4A,B.

Second, we also tested whether the IOP could be reduced further by altering the vacuum to 500 mbar from 700 mbar together with reduced surgical compression as above. This also resulted in a significant reduction compared to standard procedure (83.22 ± 13.93, p < 0.0001). Although there was no overall reduction over reduced compression (p = 0.33), there was a further reduction in the laser cutting stages (c^1^ to e^1^), between 83–95 mmHg, p < 0.01. Representative plots showing the differences in IOP during Z6 LASIK are shown in Fig. 4C by reduction in compression alone, reduction in vacuum to 500 mbar and reduced compression without suction.

### The effects of angulation on IOP

As the effect of suction could not be safely reduced for the Z8 and compression had already been neutralised, we next investigated if abrogating the effect of the weighted hand piece can further reduce IOP. It should be noted here that the moveable arm on the LDV Z8 platform has a counterweight so as to minimize the effect of the hand piece weight.

**Figure 5:**
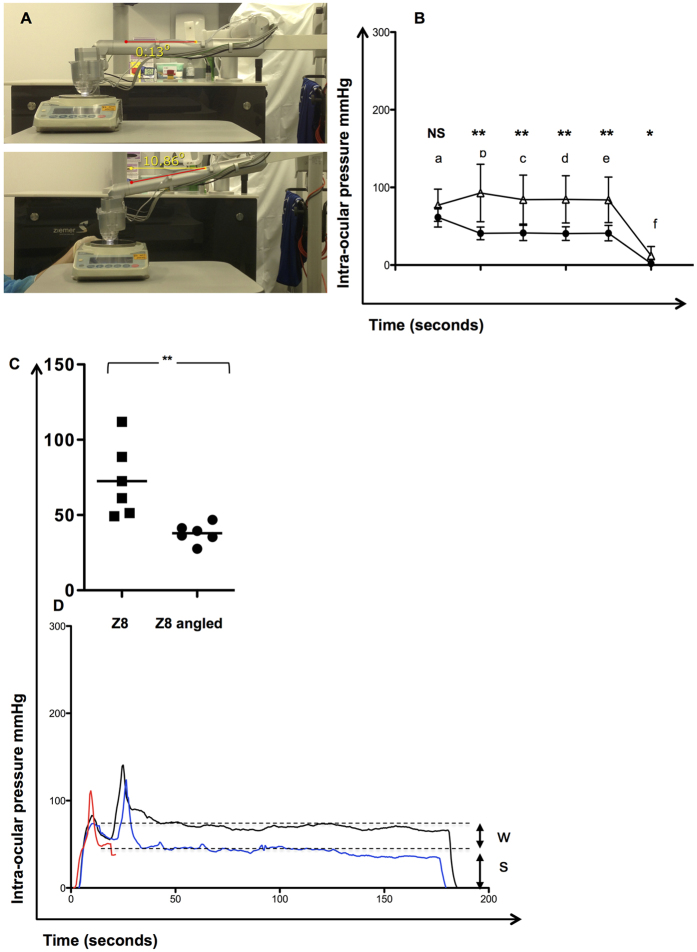
The effects of reducing the counterweight (cantilever) and surgical compression on intra-ocular pressure during Ziemer Z8 fragmentation and capsulotomy. The weight of the laser handpiece is reduced by a supporting weight acting as a cantilever, with a manufacturers angulation recommended at 0°. Decreasing the angulation has the effect of increasing the effective lift on the handpiece. Colour photograph showing the angulation of the Ziemer LDV Z8 arm with hand piece positioned on weighing scale to determine the contribution of angulation on the effective weight on the globe. The effective weight at ≈0° and −10° were 310 and 110 g respectively (panel (**A**)). Comparison for individual stage and overall IOP between the conventional 700 mbar Z8 settings at both 0° (open triangles) and −10° angulation (closed circles) (panel (**B**)) and as an average (panel (**C**)) are shown. The sequence of the procedure is: Time ‘0’; a Applanation*; b Vacuum followed by liquid filling and attachment of laser hand piece, corresponding to the peak IOP (Z8); c Lens Fragmentation; d Pause; e Lens Capsulotomy; f Cessation of suction and normalization of IOP. Analysis was by the unpaired t test (p < 0.05 significant). (Representative plots showing the differences in IOP during Z8 Fragmentation/Capsulotomy in D by conventional angulation (black), reduced angulation (blue) and the hand piece alone (no suction) for comparison (red). s = suction and w = weight.*NB - the globe was compressed with the applanation device prior to the generation of adequate suction. Comparison is by the unpaired t test (p < 0.05 significant). p < 0.01**, p < 0.05*.

At a recommended angulation close to zero degrees, the hand piece was measured at 310 g (Fig. 5A**, upper panel**). By reducing the angulation to −10°, the weight was reduced to 110 g due to the cantilever effect of the counterweight on the laser arm (Fig. 5B, **lower panel**). In turn this resulted in a measurable overall reduction in the IOP exerted on the porcine globe from 72.5 mmHg (±24.2) to 36.4 mmHg (±7.7), (Fig. 5B,C), p = 0.006. A representative example is seen in Fig. 5D and compared to the IOP without the use of suction.

### Assessment of pre-suction and post laser IOP in human subjects

The IOP measurements at baseline were measured at 12.3 mmHg (±3.71) and 42.5 mmhg (±6.52) following suction in a cohort of healthy individuals undergoing routine femtosecond laser cataract with the LDV Z8 (n = 10). This represented a mean escalation in IOP of 30.20 mmHg ± 7.50 (range 19.0–41.0 mmHg).

## Discussion

We have demonstrated through a porcine *in vivo* model that IOP with the Ziemer LDV femtosecond laser was lower using the liquid patient interface compared to the flat applanation system. Several factors played a part in the generation of IOP including the effect of the surgeon compressing the globe to help achieve suction, the effect of suction itself via vacuum and the weight of the laser system’s hand piece.

There is evidence that much higher IOP is generated using a flat applanation interface for docking, in the region of 135–205 mmHg with the Intralase and older LDV systems (184 mmHg), compared to curved systems such as the Visumax at 65 mmHg^5^,^6^,^11^. Comparable differences have been observed using human cadaveric eyes^2^, and we also previously showed that similar figures were generated in a live rabbit model using the Visumax platform, measuring a peak IOP of 83 mmHg (corresponding to stage b^1^), with the same Labchart anterior chamber cannulation device^12^. An increase of 2.7 mmHg per 100 μm increase in central corneal thickness has recently been described with the use of a tonometer^13^. It is possible therefore that the thicker cornea seen in this porcine system, known to have an average thickness of 630 μm, may have over-estimated the IOP compared to human corneas which are thinner at 530 μm^14^,^15^.

It is likely that the flat interface causes mechanical compression of the cornea, resulting in higher IOP than with a curved interface^16^. In our experimental model, we showed that IOP could be reduced by the amount of pressure exerted by the surgeon. Fear of suction-loss presumably plays a role in maintaining compression following the initial docking procedure but we found that once suction had been established, we could safely neutralize the compression without suction breaks occurring. The effect of suction was calculated to be in the region of 65 mmHg with the flat interface but the IOP could be improved further by reducing hand piece vacuum to 500 mbar. Although the elevation in IOP has been attributed to suction generation (which subsequently normalizes following release)^16^,^17^ it appears from our data that there is a more complex interaction of compression and the weight exerted by the hand piece/laser head.

High pressure may cause patient discomfort, sub-conjunctival haemorrhage, and decompression sequelae such as vitreous, retinal and choroidal detachments, acute ischemic optic neuropathy or glaucomatous nerve progression^4^,^17^. The duration of the LASIK procedure is relatively short however and the risk of vascular complications low^18^. The Ziemer LDV Z8 FLACS system offers a flat applanation system like the Z6 for cornea and a liquid patient interface for cataract surgery. The latter is designed to minimize IOP rise during surgery, avoid posterior corneal folds and relies on a vacuum – fluid filling – docking system via a suction ring by negating compression. Theoretical advantages include a more gentle procedure with reduced redness and conjunctival haemorrhage^16^. The potential for complications arising from sustained, albeit relatively lower IOP, during cataract surgery raises concern in an older population. The time difference was significantly longer than for LASIK^7^,^19^ and also increased with different capsule sizes but may have been exaggerated by difficulty in imaging software detection of pupil margin and deeper posterior capsule in the porcine cadaveric eye.

Although IOP has been quantified in patients at different time-points during FLACS, to our knowledge, continuous anterior-chamber pressure recording has not been undertaken in any laser system. Existing literature suggests comparatively modest pressure rises following the suction process^7^,^8^,^20^,^21^. The LenSX system has shown an IOP rise up to 40 mmHg while the Victus platform, generates a rise of 42 mmHg during the applanation phase^9^,^20^. The Catalys liquid interface was found to cause a pressure rise of 25.9–28.9 mmHg after suction ring applanation in two separate patient studies, with a post-procedure rise of up to 36 mmHg^4^,^5^. These findings were observed by Talamo and colleagues who undertook real time comparison of the vitreous cavity in porcine and human cadaveric globes between liquid interface and curved interfaces with the Catalys system. Although each stage of the procedure was not outlined, they found that at a vacuum of 500 mbar, the mean IOP during suction was 16.6 vs. 80.4 mmHg when comparing liquid and curved interfaces^16^.

An intra-ocular cannulation device utilizing a blood pressure (BP) transducer has previously been used in porcine systems to record real-time IOP during LASIK^22^. They found that with the Intralase system, IOP reached 119 mmHg during cutting in a porcine eye compared to 62 mmHg in a rabbit eye with a Visumax system^12^. More recently a similar BP transducer was also used to measure stages at incremental stages of FLACS using the Catalys system, demonstrating a significant rise in IOP during the suction phase^23^. Direct comparisons between platforms and cannulation devices have not been undertaken however but in this manuscript we have shown how a flat interface causes significant rise in comparison to a liquid one. A curved interface represents a compromise between distortion of the cornea and associated pressure rises and a full liquid interface^24^.

To date, the coupling effect of the laser head to the suction docking effect on IOP has not been evaluated or documented with other laser systems. While broadly results appear comparable with curved (LenSX, Victus) and liquid (Catalys, Z8) platforms, it is possible that taking a tonometry reading at a stage prior to the completion of docking by bringing the laser head into contact with the suction device underestimates true IOP peak per procedure. Direct comparisons are therefore challenging. Indeed, the IOP seen after the liquid interface suction was completed, reached a mean rise of 30 mmHg in our human evaluation, similar to our porcine data with angulation at −10°. This cantilever effect may facilitate an alteration in the counterweight currently employed (measured at 5 kg). Our calculations suggest that an additional weight in the region of 1.5 kg also effectively neutralized the hand piece weight and may represent a way for further reducing the IOP.

Reduction in phacoemulsification time is a key advantage afforded by femtosecond fragmentation during FLACS. The pressure rise may be variable and significant with conventional phacoemulsification^25^,^26^,^27^,^28^,^29^,^30^. Cadaveric phacoemulsification models have shown that highest pressures were recorded during hydro dissection with peak IOPs reaching 223 mmHg^27^,^28^ Other recent studies using intra-vitreal cannulation systems have shown that the peak intra-operative IOP may be lower, in the region of 40–70 mmHg^29^,^30^. The duration of the procedure where IOP was maintained above 60 mmHg varied from 48–85% of procedure time, ranging from 9 to 12.5 minutes^27^. Femto-docking therefore represents an additional exposure to high IOP. It was interesting to note that the area under the curve was measured at 3269 mmHg•seconds with the Z8 (without angulation) and 2015 mmHg•seconds with the Z6 (without accounting for surgical compression). Therefore the attrition may be similar even in the context of a shorter but higher IOP with a flat interface for LASIK.

Patients at higher risk from pressure fluctuation during surgery represent a potential contra-indication when considering FLACS. Not only does the peak pressure have to be considered, the variation during different stages of surgery and the length of the procedure in the context of identifiable risk factors should be under consideration as well. While no complications were seen in our patient cohort a longitudinal study quantifying clinical parameters of vision and optic nerve function combined with objective assessment e.g. by OCT, doppler analysis and field evaluation in healthy individuals would add further insight in to the effects of IOP in FLACS. In this paper we were able to demonstrate that IOP was considerably higher with a flat vs. liquid interface, and this could be relieved by various factors including the effect of the surgeon compressing the globe, the vacuum pressure with the flat interface and the angulation with the Z8 to a level that we would consider a safer IOP threshold during FLACS.

## Methods

### Porcine *ex vivo* Model

An *ex vivo* model to determine real-time intra-ocular pressure change was undertaken as previously described^12^,^31^. Briefly, porcine eyes were recovered and used within six hours of retrieval from a local abattoir. Eyes were mounted on a pressurized stand and a 30-gauge cannula connected to an IOP catheter transducer was inserted into the anterior chamber, posterior to the limbus (Fig. 1A,B). The LabChart 6 (ADI Instruments, Dunedin, New Zealand 2008) transducer was used according to manufacturer’s instructions and calibration was performed before starting each trephination. To ensure the transducer was working accurately, IOP measurements were also taken from a sample of the first 18 eyes with a Tonopen (Reichert-Jung, Depew, NY, USA) (r = 0.98, p < 0.0001) (Fig. 1C).

Each procedure was carried out with standard clinical settings for lens fragmentation (8 segment, 5.3 mm diameter, 2.8 mm height, cut speed 10 mm/s, power 100%, Repetition rate 2 MHz, Pulse duration 250 fs) and capsulotomy (4, 5 or 6 mm diameter, 0.8 mm height, cut speed 50 mm/s, Repetition rate 1 MHz, Pulse duration 250 fs, power 90%) with the LDV Z8 (software version X.5054). LASIK flap creation with the LDV Z6 system (Ziemer, Port, Switzerland 2014) (z-Lasik z 8.8 mm diameter, 110 μm height, cut speed 14.5 mm/s, Repetition rate 10 MHz, Pulse duration 250 fs, stroma energy 95%).

The following comparable time points during both separate procedures were documented during continuous recording of IOP from Time ‘0’ through docking (applanation and suction generated by vacuum) and procedure: a Applanation* (a Liquid patient interface, Z8 or a^1^ Flat applanation, Z6); b Vacuum/Filling with liquid/Attachment of laser or b^1^ Vacuum alone; c Fragmentation (c^1^ Lamella cut); d Pause (d^1^ Pause); e Capsulotomy (e^1^ Side cut); f/f^1^ Cessation of suction and normalization of IOP.

*NB - the globe was compressed with the applanation device prior to the generation of adequate suction and allowed to attain a neutral position or one that maintained suction during the procedure.

Measurements were also undertaken without the application of suction and by reducing surgical compression by the hand piece and/or by abrogating the weight of the hand piece arm counterweight by altering the angle of contact with the eye. Further comparisons were made between high and low-pressure Z8 system settings (400 mbar manufacturers recommended setting and reduced 300 mbar setting) and with the Z6 LASIK system settings (700 mbar manufacturers recommended setting and 500 mbar setting).

### Human Study

Patients undergoing routine Femtosecond cataract surgery with the Ziemer LDV Z8 system at the Oftalmosalud Instituto de Ojos (LI) had Tonometry undertaken from the central cornea at the pre-docking (analogous to pre-cannulation in the porcine study), docking (analogous to stage a) (prior to the coupling of the laser handpece, middle panel Fig. 1B) and post laser stages of surgery (analogous to post-cannulation) as part of a service evaluation and in accordance with approved guidelines. Patients had a median age of 73.2 years [range 69–77], 8 were Caucasian and 2 Hispanic and did not have glaucoma or other co-existing pathology prior to surgery. All patients treated conformed to the tenets of the declaration of Helsinki and committee approval for measurements to take place as a service evaluation was granted by the Oftalmosalud Instituto de Ojos, Lima, Peru. Written consent was obtained from all patients.

### Statistical Analysis

Data was analysed by Excel v15.0 for Windows (Microsoft, Redmond, WA, USA) and comparisons were undertaken by non-paired t-tests, Analysis of Variance (ANOVA) with Bonferonni’s post hoc test and Pearson correlations using Prism version 5.0 for Macintosh (GraphPad Software, La Jolla, CA, USA) with p < 0.05 deemed to be indicative of statistical significance. Data is expressed as mean ± SD unless stated.

## Additional Information

**How to cite this article**: Williams, G. P. *et al.* Comparison of intra-ocular pressure changes with liquid or flat applanation interfaces in a femtosecond laser platform. *Sci. Rep.* **5**, 14742; doi: 10.1038/srep14742 (2015).

## Supplementary Material

Supplementary Information
