# Supplementary material for: Comparison of intra-ocular pressure changes with liquid or flat applanation interfaces in a femtosecond laser platform
Source: Sci Rep. 2015 Oct 6;5:14742. doi: 10.1038/srep14742 (PMC4593965; doi:10.1038/srep14742)
Supplement: Supplementary Information [file srep14742-s1.pdf]

Comparison of intra-ocular pressure changes with liquid or flat applanation interfaces in a femtosecond laser platform

Williams GP [1,2], Ang HP [1], George BL [1], Liu YC [1], Peh G [1], Izquierdo L[6], Tan DT [1,2,3,4] and Mehta JS [1,2,4,5*]

[1]Tissue Engineering and Stem Cell Group, Singapore Eye Research Institute, Singapore

[2]Singapore National Eye Centre, Singapore

[3]Department of Ophthalmology, Yong Loo Lin School of Medicine, National University of Singapore, Singapore
[4]Ophthalmology Academic Clinical Program, Duke-NUS Graduate Medical School, Singapore

[5]Department of Clinical Sciences, Duke-NUS Graduate Medical School, Singapore
[6] Oftalmosalud Instituto de Ojos, Lima, Peru

Supplementary Information

Supplementary Figure 1

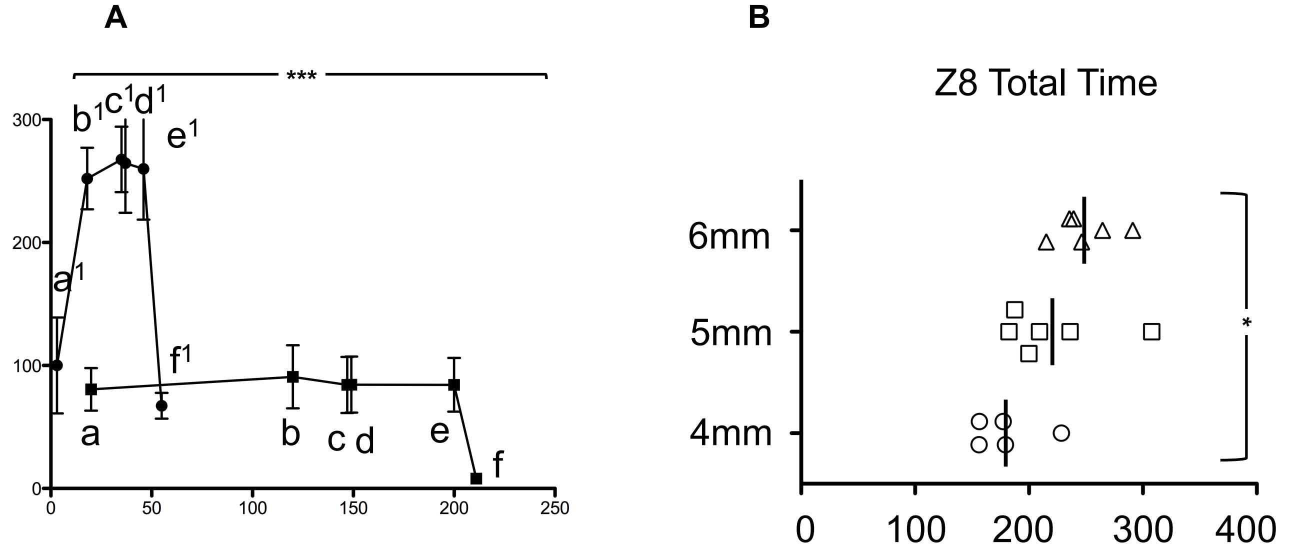

Figure S1. Procedure time with the Z8 and Z6 laser platforms for capsulotomy/ fragmentation and LASIK and taken to undertake different capsulotomies.
Panel A shows a schematic representation of the timeline during femtosecond fragmentation/ capsulotomy with the Ziemer LDV Z8 and LASIK with the LDV Z6 (Panel A). The time taken to complete different capsulotomies is shown in panel B. Comparison is by students t-test or Analysis of Variance with Bonferonni correction ($p<0.05$ significant).

Supplementary Tables

Procedure	Total Time			Average IOP			Average IOP		
	Z8 ¶	Z6 §	Significance	Z8 ¶	Z6 §	Significance	*Z8 angled ¶*	*Z6 reduced compression §*	*Significance*
Docking: a Suction ring application or a[1] Applanation	20.9 (3.3)	4.0 (2.9)	**p<0.0001**	77.1 (20.7)	100.1 (39.0)	p=0.23	61.5 (12.5)	61.5 (19.5)	p=0.97
b/b[1] Suction	106.8 (44.8)	15.4 (4.2)	**p=0.0006**	92.8 (37.1)	252.0 (25.0)	**p<0.001**	40.8 (8.2)	129.6 (20.1)	**p<0.0001**
c Fragmentation/ c[1] Lamella cut	26.2 (0.4)	17.2 (2.9)	**p<0.0001**	84.3 (31.6)	267.6 (26.6)	**p<0.0001**	41.3 (9.9)	109.8 (5.2)	**p<0.0001**
d/d1 Pause (Interval between Stages c/c[1] & e/e[1])	4.4 (0.3)	1.7 (0.3)	**p<0.0001**	84.6 (30.4)	264.5 (40.4)	**p<0.0001**	40.5 (8.8)	108.2 (5.9)	**p<0.0001**
e Capsulotomy e[1] Side cut	50.7 (0.3)	8.7 (1.8)	**p<0.0001**	84.0 (29.3)	259.9 (41.3)	**p<0.0001**	41.0 (9.9)	106.6 (6.9)	**p<0.0001**
f/f[1] Removal of interface and normalization of IOP	11.5 (1.4)	9.1 (1.4)	**p=0.02**	12.2 (11.8)	67.3 (10.4)	**p<0.0001**	2.9 (4.0)	22.8 (4.0)	**p<0.0001**
Complete procedure	220.4 (46.8)	56.1 (2.9)	**p<0.0001**	72.5 (24.2)	201.9 (18.6)	**p<0.0001**	37.8 (6.5)	89.7 (7.1)	**p<0.0001**

Table S1: Summary table of time and intra-ocular pressure (IOP) with the Ziemer LDV Z8 and Z6 platforms to undertake fragmentation/capsulotomy and LASIK flap creation respectively under different conditions

Values shown are mean with standard deviation

Significance was taken at p<0.05 (Unpaired t test)

¶ Liquid interface system (n=6)

§ Curved contact applanation system (n=6)

a Docking (a Suction ring application or a[1] Applanation); b/b[1] Suction; c Fragmentation (c[1] Lamella cut); d Pause (d[1] Pause); e Capsulotomy (e[1] Side cut); f/f[1] Normalization of IOP).
